# Neuroinflammation and Precision Medicine in Pediatric Neurocritical Care: Multi-Modal Monitoring of Immunometabolic Dysfunction

**DOI:** 10.3390/ijms21239155

**Published:** 2020-12-01

**Authors:** Kristine E. Woodward, Pauline de Jesus, Michael J. Esser

**Affiliations:** Alberta Children’s Hospital, University of Calgary, Calgary, AB T3B 6A8, Canada; kristine.woodward@ahs.ca (K.E.W.); pdejesus@ucalgary.ca (P.d.J.)

**Keywords:** neuroinflammation, neurocritical care, pediatric, multi-modal, precision medicine

## Abstract

The understanding of molecular biology in neurocritical care (NCC) is expanding rapidly and recognizing the important contribution of neuroinflammation, specifically changes in immunometabolism, towards pathological disease processes encountered across all illnesses in the NCC. Additionally, the importance of individualized inflammatory responses has been emphasized, acknowledging that not all individuals have the same mechanisms contributing towards their presentation. By understanding cellular processes that drive disease, we can make better personalized therapy decisions to improve patient outcomes. While the understanding of these cellular processes is evolving, the ability to measure such cellular responses at bedside to make acute care decisions is lacking. In this overview, we review cellular mechanisms involved in pathological neuroinflammation with a focus on immunometabolic dysfunction and review non-invasive bedside tools that have the potential to measure indirect and direct markers of shifts in cellular metabolism related to neuroinflammation. These tools include near-infrared spectroscopy, transcranial doppler, elastography, electroencephalography, magnetic resonance imaging and spectroscopy, and cytokine analysis. Additionally, we review the importance of genetic testing in providing information about unique metabolic profiles to guide individualized interpretation of bedside data. Together in tandem, these modalities have the potential to provide real time information and guide more informed treatment decisions.

## 1. Introduction

The study of neuroinflammation in the critically ill is a broad field that is rapidly evolving. Neuroinflammation can be broadly thought of as comprising the physiological, biochemical, and cellular reactions to primary or secondary insults to nervous system tissue (brain, spinal cord, and autonomic nervous system). Examples of primary injury include autoimmune encephalitis, primary CNS angiitis, and primary demyelination, whereas secondary neuroinflammation can occur as a result of infection, traumatic brain injury, and toxic metabolites, and potentially many other neurological and systemic insults [1]. While neuroinflammation can occur as a normal biological response to protect the brain parenchyma, if unregulated it can result in severe neurological compromise and poor outcomes. Most patients admitted to a pediatric intensive care unit (PICU) with a critical illness may be at risk for some degree of neuroinflammation, but what is not clear is what component of the response is beneficial versus harmful. It is this question that makes the study of neuroinflammation and immune dysfunction an important area of research, with the potential to improve acute and chronic outcomes as well as patient quality of life. While previously treatment was targeted towards inhibition of all inflammation, efforts have now shifted to a better biological understanding and acknowledgement that not all inflammation is detrimental, and there may be more benefit in targeted or selective modulation of inflammation [2]. Ongoing research efforts are also better ascertaining integral metabolic pathways associated with neuroinflammation, and characterizing both beneficial and detrimental effects, as well as the dynamic nature of these molecular mechanisms [3]. Mapping the immunometabolic and inflammatory process over time offers the potential to conceptualize more precise treatment strategies by providing specific and changing “targets” throughout the clinical course. However, this is an oversimplification of a highly dynamic, intertwined network that is compounded by significant inter-personal variability depending on a plethora of individualized factors. Ideally, real-time bedside monitoring of neuroinflammation itself, or the functional impact on the CNS, could afford the opportunity to map changes in individualized neuroinflammatory responses to guide precise, personalized management.

This paper aims to review the basics of CNS immunometabolism and its fundamental role in neuroinflammation as it pertains to CNS dysfunction, and introduces bedside monitoring tools amenable to critical care settings to provide indirect measures of immunometabolic shifts and resultant inflammation. Genomic data is an important link within this construct to provide information about the unique metabolic profile of patients and may change interpretation of bedside data and therapeutic strategies. Ultimately, in combination, these tools have the potential to be used in a multi-modal manner to guide real-time individualized treatment.

## 2. CNS Neuroinflammation and Immunometabolism

Inflammation is a complex biological response of the body to cell and tissue damage. The degree of inflammation is dependent on the type of injury and extent of tissue affected. Controlled inflammation facilitates cell repair, turnover, and protection from the source of damage [4]. However, when unregulated, unchecked and persistent inflammation can result in further cell damage, and poor long term outcomes [4]. When an inflammatory cascade is triggered, immune cells shift from a resting to activated state, and alter their primary mechanism of energy metabolism [3]. This dynamic cell metabolism and resultant immunological functioning, or immunometabolism, is a growing area of research. While most research to date has focused on peripheral responses, specifically macrophage immunometabolism, new efforts have been made to determine whether the same type of re-programming occurs in the central nervous system, specifically within microglia and astrocytes, in response to neuroinflammation as a consequence of injury [3]. This phenomenon, or change in glial cells in response to their environment, is called reactive gliosis. This facilitates rapid changes in cell morphology, gene expression, secretory profiles, which necessitates a change in cellular metabolism [5].

**Figure 1:**
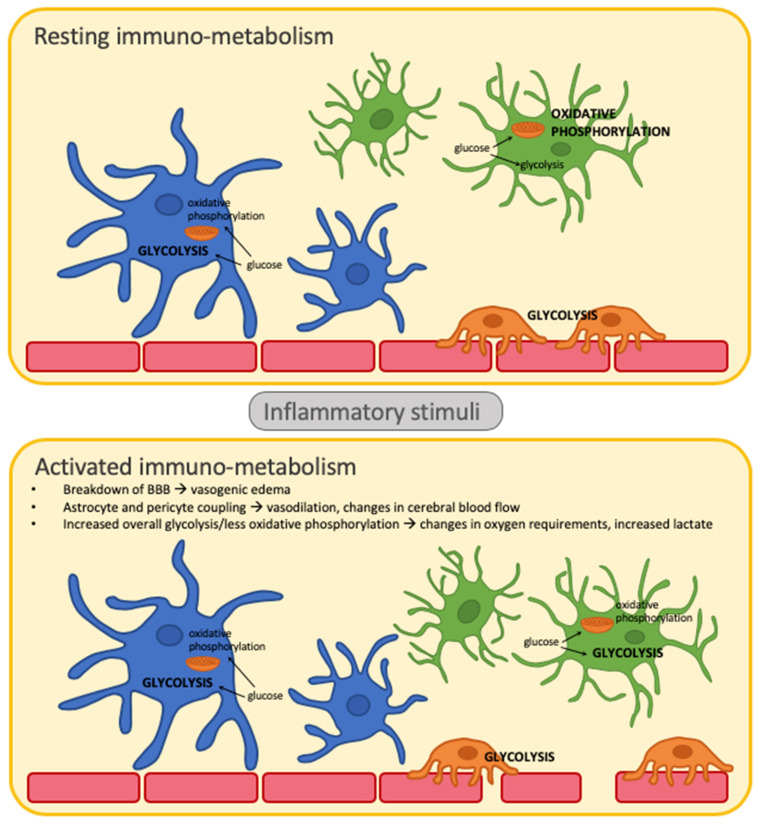
Immunometabolism of CNS immune cells at rest and in response to inflammatory stimuli. Blue cells are astrocytes, green cells are microglia, orange cells are pericytes, and red cells are the endothelial cells lining vasculature. Bolded and capitalized letters indicate the primary mode of cellular metabolism during either the resting or activated state. Astrocytes rely on glycolysis during rest and continue to use this as their primary mode of metabolism when activated by an inflammatory trigger, however in an upregulated manner. Pericytes also use glycolysis for energy production during both resting and activated states. Microglia metabolize using oxidative phosphorylation during rest, and switch to glycolysis during activated states.

During inactivated states, microglia “survey” the environment to maintain cellular homeostasis, an action that requires constant cytoskeletal rearrangement, which is very energy demanding [6]. In order to meet these significant energy demands, microglia rely on mitochondrial oxidative phosphorylation as this produces a significant amount of ATP (Figure 1) [3,7]. If there is a neuroinflammatory signal in response to primary or secondary stimuli, microglia shift their metabolic pathway towards glycolysis (Figure 1) [3,7]. Glycolytic end products are then shuttled into the pentose phosphate pathway (PPP) or sent for lactate production [8]. The PPP provides cellular building blocks, including nucleotides, proteins, and lipids, which are necessary for increased cell growth and proliferation, as well as lactate which provides neurons with an alternative energy source [9]. This metabolic pathway can be completed with limited oxygen, a typical consequence of an acute neurological injury and the often associated neuroinflammatory cascade.

At rest, astrocytes are responsible for maintaining homeostasis within the synaptic environment [3]. They rely primarily on glycolysis to make lactate as a neuronal energy source and glutamine for neurotransmitter synthesis (Figure 1). Once microglia are activated, they can in turn activate astrocytes [3,6]. Research regarding metabolic shifts in astrocyte metabolism is limited compared to other immune modulators, however it is believed that activated astrocytes continue utilizing glycolysis (Figure 1) [6,10]. Glycolytic metabolism is upregulated in order to meet the increased energy demands and is preferable given its competency in anaerobic conditions.

Although neurons are not considered part of the immune system, their metabolic activity contributes to the local and paracrine biochemical environment, thereby potentially impacting the function and activity of local immune cells. Being highly energy demanding, neurons are primarily driven by oxidative phosphorylation in order to produce a significant amount of ATP through the mitochondrial electron transport chain [10]. However, during times of oxygen deprivation, neurons can also utilize lactate as an energy source [8]. This is an area of significant debate, and there are arguments regarding whether this may be a preferential energy source even when there is ample oxygen supply [11].

The immunometabolic environment also plays a role in neurovascular coupling. The neurovascular unit consists of neurons, interneurons, astrocytes, microglia, and their interaction with pericytes on capillaries, vascular smooth muscle cells on arterioles and arteries, and endothelial cells [12]. The interplay of cells in the neurovascular unit contributes to modulation of cerebral blood flow (CBF) as well as blood–brain-barrier (BBB) permeability, depending on the surrounding parenchymal milieu, and particularly whether there is concurrent inflammation [13]. Neuroinflammatory mediators (e.g., cytokines, chemokines, growth factors, damage-associated molecular patterns (DAMPs), substance P, neurokinins, matrix metalloproteinases) [6] generally cause increased permeability, and secondary effects of this including vasogenic edema, or extracellular accumulation of fluid and extravasation of serum proteins (Figure 1) [14]. Pericytes are potent responders to surrounding neuroinflammation, and can constrict or dilate around vasculature to help regulate cerebral blood flow and match energy demands [15]. They also are important gate-keepers along the BBB together with endothelial cells and astrocyte end-feet, forming a tight barrier to peripheral cells [6]. Their role changes in response to cytokines released during neuroinflammation, causing changes in CBF and BBB permeability [16]. Such changes are facilitated through immunometabolic shifts and upregulation of baseline glycolysis to meet increased energy demands [3,6]. Energy metabolism in pericytes is driven by glycolysis to provide optimal functioning in oxygen deprived states, but it is still the primary source of energy during quiescent periods (Figure 1) [17].

Immunometabolic shifts during neuroinflammatory states hypothetically function to provide support for cellular protection by promoting clearance of debris and cellular regeneration. However, dysregulated neuroinflammation and dysfunctional immunometabolism can ultimately cause secondary injury through cellular damage, dysfunction, or death (in neurons, astrocytes, microglia etc.), potentiating neurodegeneration [18]. Unregulated neuroinflammation is correlated with substantial release of proinflammatory cytokines, and together with poor oxygen delivery, causes increased and excess production of harmful intermediates such as mitochondrial reactive oxygen species (ROS) [19]. ROS are produced when oxygen is only partially reduced through the electron transport chain in the mitochondria during oxidative phosphorylation. While more evidence points towards benefit in small numbers, ROS in excess can be harmful and cause significant cellular damage as well as cellular apoptosis [20]. The most common cellular free radicals are hydroxyl, superoxide, and nitric monoxide. They induce cellular injury in the form of lipid peroxidation, DNA damage, and protein peroxidation, eventually leading to cellular apoptosis [21]. The classical mechanism by which this occurs is via cytochrome c oxidase (CCO) release, the terminal electron acceptor in the electron transport chain. In normal conditions it acts as the rate limiting step of the respiratory chain, and its activity is an indicator of the oxidative capacity of the cell [20]. As such, CCO serves as a measure of functional oxidative phosphorylation within the cell. CCO is also involved in the initiation of apoptosis. ROS migrate through the mitochondrial membrane, mobilize CCO into the cytosol, and form an apoptosome. This then activates initiator caspase 9 and 3 to potentiate cell death [22]. This mechanism is particularly detrimental within the BBB endothelium, causing permeability within the tight junctions and allowing passive flow of toxic metabolites. In particular, ROS cause altered expression of critical tight junction proteins such as claudin-5 and occludin [21]. Endothelial and consequently vascular injury via ROS also results in poor neurovascular coupling. Mechanisms include activated microglia increasing ROS production, leading to pericyte apoptosis through caspase mechanisms. Additionally, ROS such as superoxide lead to increased Rho kinase signaling, interfering with endothelial nitric oxide synthase expression and activity, affecting nitric oxide production and inducing both abnormal vasodilation and vasoconstriction of the cerebral vasculature [21]. ROS have many more contributions to neuronal functioning, but these are some of the most common mechanisms, to name a few. Other contributions to the inflammatory cascade include excess release of glutamate, and other excitatory amino acids, further potentiating metabolic strain during excitotoxicity and hypoxia, and thereby secondary energy failure and further oxidative failure including ROS [20].

Cytokine activity is involved in a complex interplay between cell protection, proliferation, and apoptosis, given its dual participation in activation and inhibition of cellular components. Classic neuroinflammatory diseases are triggered when the CNS is invaded by blood-borne leukocytes, triggering cytokine proliferation and release. Initial release of key cytokines includes IL-1β, IL-6 and TNFα, which are produced by CNS-resident cells, particularly T helper cells [23]. IL-12 and IL-23 are produced by antigen presenting cells and influence differentiating T cells towards their ultimate phenotypes. Signaling by IL-23 promotes downstream production of IL-17, IL-22 and IL-23 [23]. IL-17 and IFN-gamma that can have a significant effect on the BBB endothelium, disrupting its integrity and allowing leukocytes to cross into the CNS parenchyma [23]. This can then potentiate ongoing inflammatory infiltration and resultant parenchymal damage. GM-CSF causes monocytic shifts towards pro-inflammatory states and results in them becoming highly phagocytic and destructive [23]. Importantly, many of these cytokines are markers of neuroinflammation and endothelial damage. To complete the circle, these cells also release ROS that can further potentiate inflammation. Cytokines continue to be a multifaceted area of study, and further research is required to fully understand their complexity and dual nature in the pathogenesis of neuroinflammation.

Pediatric patients introduce yet another variable into the dynamic process of CNS cellular immunometabolism given the variations in metabolic demand that occurs during development. Fundamentally, brain development tends to be evaluated from a neuron centric perspective with emphasis on factors and modulators of neurogenesis, organization, and function. However, microglia and astrocytes also play an important role during brain development. They are involved in neurogenesis and gliogenesis, axonal growth, angiogenesis, synaptic formation and pruning, thereby contributing to the increased metabolic demand during this time period [24]. During early childhood, there is a 2.5-fold increase in the global cerebral metabolic rate of glucose, a 1.5-fold increase in the global cerebral metabolic rate of oxygen, and a 2-fold increase in global cerebral blood flow [25]. The course over which these changes occur is variable, both temporally and spatially, leading to differences in metabolic reserve during primary and secondary injury, and response to therapeutic targets. Individualized, and dynamic monitoring techniques of metabolic changes would eliminate the need for estimating values based on age during the dynamic process of neural development.

In summary, neuroinflammation is highly dependent on the metabolic capacity of the various cells making up the immune response during injury, which alters the composition of the surrounding environment. Changes in metabolism affect oxygenation, vasoreactivity, and lactate production, which are indices that can be monitored using non-invasive bedside techniques. This milieu changes based on the composition of activated vs resting state of resident neurons, glia, and immune cells, and thereby has the potential to serve as secondary markers of beneficial vs. detrimental inflammation. Efforts to balance metabolic demands that occur during neuroinflammation due to shifts in immunometabolism can be targeted towards decreasing significant energy demands or augmenting cellular protective mechanisms in the face of the increased energy demands (i.e., reducing generation of reactive species) and avoiding hypoperfusion and hypoxia. With high temporal and spatial resolution monitors guiding treatment decisions, we may be able to intervene more effectively during neuroinflammatory states at earlier time points, that are specific to each individual, to prevent both further acute damage and chronic harmful inflammatory states.

In the following sections we will review different modalities that can aid in improving the fundamental understanding of brain function and pathophysiological mechanisms impacting brain health. These tools are amenable to evaluating immunometabolism, albeit indirectly, and offering the potential of personalized bedside management.

## 3. Non-Invasive Monitoring

### 3.1. Near Infrared Spectroscopy (NIRS)

NIRS is a non-invasive bedside tool that can provide continuous monitoring of local brain oxygenation, hemodynamics, and metabolism. NIRS has a wavelength of 700–900 nm which can easily penetrate living organisms [26]. Light absorption in this wavelength occurs mostly by deoxyHb and oxyHb, both having different absorption spectra. Depending on oxygenation status, hemoglobin scatters and absorbs varying amounts of light thereby changing the emitting spectra, which is detected by the NIRS sensor to determine tissue oxygenation [26]. As mentioned, the primary chromophores used in NIRS are oxyhemoglobin and deoxyhemoglobin. However, in addition, a unique copper dimer (Copper A) in cytochrome-c-oxidase (CCO) is a chromophore that is a marker of cellular oxygenation and energy metabolism. CCO is the terminal electron acceptor of the electron transport chain within mitochondria, which is responsible for 95% of cellular oxygen metabolism [27]. NIRS measures the spectral difference between the oxidized CCO and reduced CCO, thereby providing a more direct, albeit still secondary, measure of oxidative phosphorylation [27]. Given these properties, NIRS has the potential to monitor, in vivo, dynamic changes in oxygenation, metabolism, and local cerebral hemodynamics.

Currently, NIRS use in NCC is primarily focused on detecting changes in tissue oxygenation and metabolism in patients with cerebrovascular disease, hypoxic ischemic encephalopathy (HIE), and traumatic brain injury. In all of these conditions, or insults, there is a component of secondary neuroinflammation following the inciting event. In ischemic stroke, cellular injury cascades secondary to a loss of glucose and oxygen trigger inflammatory signals that can result in changes in microvasculature and BBB disruption, and later secondary excitotoxicity from ROS [28]. This causes neuronal injury in the acute period, as well as potential accelerated long term neurodegeneration from chronic inflammation. Studies using NIRS have been able to map this process and have shown that oxygenation and metabolism change over time depending on the extent of injury after acute ischemic stroke [29]. Infarcted brain has near normal oxygen levels, if not elevated, presumably due to lack of utilization, whereas ischemic brain tissue that has not yet infarcted has low oxygenation due to significant utilization from increased metabolic demand [30]. These findings provide a framework supporting the utility of being able to earlier identify “brain at risk”, and potentially intervene sooner to protect vulnerable tissue [31]. Studies complementing this have shown reduction in tissue oxygenation when comparing pre and post recanalization following large vessel occlusion [32], suggesting increased metabolic demand of rescued tissue with reperfusion. Pediatric studies of perinatal stroke have further looked at direct metabolism via CCO, and shown that after injury, dynamic changes in CCO are very restricted [33]. This finding may represent mitochondrial oxidative damage, thereby indicating cellular inability to reduce and oxidize CCO through the electron transport chain in order to produce ATP, and lead to secondary energy failure and cell death [33].

In neonatal HIE, a significant mediator of poor outcomes is secondary neuroinflammation [34]. Hypothermia is used in an attempt to minimize secondary brain injury by decreasing cerebral metabolism and inflammation during the first 72 h after insult. Immunometabolic imbalance plays a significant role in HIE. Both post-mortem and animal studies of HIE show significant microglial infiltration into the brain parenchyma, which contributes to the significant secondary neuroinflammatory response [34]. Hypothermia is thought to minimize this harmful reaction. Studies using NIRS have demonstrated metabolic disruption during this time period, and similar to perinatal stroke, the state of CCO (i.e., the spectral difference between reduced and oxidized CCO) was highly dependent on, and passively fluctuated due to systemic hemodynamic variables indicating the inability to appropriately reduce or oxidize CCO due to metabolic energy failure [35]. NIRS has also been used as an indirect measure of cerebral blood flow (CBF) in HIE, where decreased CBF, as a surrogate for functioning of the neurovascular unit, may potentially provide a more accurate prognostication about the severity of HIE on the first day of life [36]. NIRS has the ability to measure the immunometabolic response via secondary indicators of cellular and tissue response, and could provide more precise time points to guide cooling windows and inform prognostication.

Traumatic brain injury is another area where the utility of NIRS continues to be actively investigated. Similar to HIE, the mainstay of treatment in TBI is preventing damage from secondary cascade mechanisms including elements of inflammation, impaired energy metabolism, structural damage, and cerebral edema. One significant contributor to poor outcomes after TBI is loss of global and regional cerebral autoregulation and cerebrovascular reactivity, thereby creating a mismatch between metabolic needs and supply [37]. NIRS can indirectly monitor cerebral autoregulation by measuring local tissue cerebral oxygen concentration and correlating this with CBF through its relationship with mean arterial pressures [38]. Further, studies have shown that tissue oxygenation index is lower in patients with elevated ICP in TBI [38], again suggesting the utility of NIRS. In addition, as NIRS is a non-invasive modality compared to the gold standard of intracranial probes [38], it could serve as a means to provide earlier, and more frequent time point assessments to help modify therapeutic interventions.

While all of these studies have used NIRS to monitor pathological changes over time, they have mostly been exploratory in an effort to better understand pathophysiology, to serve as a monitoring tool (e.g., pre and post-surgical changes), or to aid in prognostication. However, NIRS also has the potential to guide pharmacological treatments. As inflammation is thought to be a negative modulator of cellular hormesis, better ascertaining the role of immunometabolic dysregulation impacting immune cell function and subsequent inflammation could potentially drive development and implementation of pharmaceutical interventions or tailored strategies. Importantly, these changes occur at different time points, and are dynamic in individual patients, emphasizing the importance of individualized monitoring for timing of therapy implementation.

### 3.2. Ultrasound

#### 3.2.1. Transcranial Doppler (TCD)

TCD is a non-invasive technique that uses ultrasound technology to visualize tissue structure and function. A probe, or transducer, emits an ultrasonic beam that passes through the extracranial tissue and is targeted specifically towards red blood cells, which are flowing through the intracranial vasculature [39]. The ultrasonic beam is reflected by the moving red blood cell, and ultimately changes the frequency, creating the doppler shift, which is proportionally correlated to the velocity of the object [40]. Put another way, the doppler shift represents the difference between the transmitted and received signal, while the time interval from pulse emission to reception determines the depth at which the doppler shift is detected. This gives information about cerebral blood flow velocity in the location that it is being measured. These measurements can only be made through thinner areas of bone in the skull, also known as acoustic windows [39]. As such, because of proximity, some vasculature is relatively easier to measure than others including the major intracranial vessels (internal carotid artery, medial cerebral artery, anterior cerebral artery, posterior cerebral artery, vertebral arteries, and the basilar artery). These velocities can also be used to determine the pulsatility index, which is a secondary measure of vascular resistance determined by calculating the doppler frequency shifts during a cardiac cycle [39]. By measuring cerebral blood flow velocity in combination with the pulsatility index, inferences can then be made about vascular reactivity; an important component to ensuring appropriate cerebral perfusion in the face of changing cardiac output, intravascular volume and peripheral vascular tone. This then can inform on clinically important entities such as vasospasm, changes in intracranial pressure, and loss of vascular tone portending potential loss of dynamic cerebral autoregulation.

Currently in neurocritical care, TCD is used most often for monitoring and management of patients with TBI and subarachnoid hemorrhage (SAH). As mentioned previously, these cerebral insults have associated harmful acute inflammatory responses as well as potentially persistent inflammation leading to long term neurodegeneration. Similar to NIRS, studies using TCD in TBI have shown impaired cerebral autoregulation [41]. The neuroinflammatory response in TBI is particularly harmful to blood flow, as it is already compromised due to direct injury, secondary effects from cerebral edema, and impaired functioning of the neurovascular unit. This can lead to phases of early hypoperfusion, followed by hyperemia, as well as delayed cerebral vasospasm and raised ICP [42]. TCD monitors CBF by measuring middle cerebral artery velocities and pulsatility indices as a surrogate marker of ICP [43]. These measures have been shown to act as early markers of cerebral edema and raised ICP, providing a window for earlier intervention [44]. Additionally, the finding of impaired cerebral autoregulation has been correlated with worse neurological outcomes in TBI [41].

In aneurysmal subarachnoid hemorrhage, there is significant risk of secondary injury due to vasospasm and subsequent ischemia [45]. When subarachnoid blood accumulates, an inflammatory cascade is triggered to recruit local immune cells into the subarachnoid space, including microglia and macrophages [45]. These cells are ultimately trapped in the subarachnoid space due to BBB repair, and once they degranulate, release vasoactive substances (i.e., IL-6, TNF-alpha, IL-1alpha/beta) [46] causing regional and diffuse vasospasm [47]. This is one of the major causes of delayed cerebral ischemia and worse outcomes in SAH. TCD measurements can be used to detect evidence of early vasospasm, as constriction of intracerebral vasculature leads to increased flow velocities [48]. These changes precede evidence of cerebrovascular compromise via systemic hemodynamic abnormalities, providing an early time point for intervention [49].

Early research is looking into the utility of TCD in CNS vasculitis, or primary inflammation of the blood vessels. A case of primary CNS vasculitis was described in which TCD showed patchy increases in CBF velocity indicative of segmental narrowing, which correlated with conventional angiography [50]. These changes showed resolution over time with immunotherapy, and correlated with findings of normal vessel caliber on repeat angiography post treatment [50]. Similar results were found in a study of pediatric patients with cerebral vasculitis secondary to West Nile Virus. TCD velocities of the middle cerebral artery were measured and correlated well with conventional imaging modalities of vessel anatomy to monitor treatment response and guide length of treatment with steroids and cyclophosphamide [51]. Lastly, one of the most significant uses of TCD in pediatrics is to monitor vasculopathy in sickle cell disease. Stroke is a complication due to sickle cell adherence to vascular endothelium resulting in hemolysis, and subsequent inflammatory cascade, triggering both large and small vessel vasculopathy [52]. TCD is used to measure flow velocity and evidence of stenosis, and transfusion treatment guided by TCD can significantly reduce stroke risk [53].

#### 3.2.2. Elastography

Elastography is a measurement of tissue resistance or compliance, or the tendency of tissue to deform with externally applied pressure [54]. Using ultrasound techniques, two types of properties can be measured including strain and shear elastography. Strain measures deformation of tissue when external pressure is applied. Stiffer lesions deform less with pressure and thereby have lower strain [54]. Physiologic strain can be measured by determining the strain of internally applied forces such as vessel pulsation on the surrounding tissue [54]. Alternatively, an acoustic pulse can be applied by an ultrasound machine to determine strain elastography. Ultrasound receivers calculate tissue displacement by comparing the characteristics of the ultrasound beam before and after internal compression is applied, resulting in a strain map [54]. Shear elastography is similar, using an acoustic radiation force that laterally travels through tissue, ultimately calculating how quickly the shear wave travels from point A to B, or shear velocity (rather than calculating tissue displacement) [54]. Shear waves propagate faster in dense tissue compared to softer tissues [55], and shear waves also do not propagate through fluid, making them a useful tool for assessing edema [56]. Tissue elastography can be used as a non-invasive modality to monitor the secondary effects of inflammatory changes that happen following injury. Flow alteration, microvascular injury, edema, as well as tissue regeneration and reorganization including scaring and fibrosis, can all cause changes in elastography.

While purely used in research settings, ultrasound elastography has high potential utility in NCC and brain injury, as tissue stiffness provides information about intracranial edema, intracranial pressure, compliance, and changes in perfusion [57]. Neuroinflammation causes blood–brain-barrier breakdown, thereby allowing peripheral cytokines and other inflammatory markers to pass into the parenchyma, potentially impacting tissue compliance, which can be measured by strain elastography. Research has examined changes in elastography in areas of neurocritical care including TBI and stroke. In rat models, decreased tissue stiffness has been found in the ipsilateral hemisphere due to a large middle cerebral artery stroke hours following the injury, which was attributed to liquefactive necrosis and edema [56]. Contralateral stiffness increased, which was thought to be due to increased ICP [56]. Similar results in rat models of TBI show decreased stiffness ipsilateral to the injury site, while on the contralateral side there was an increase in stiffness presumably due to similar pathophysiological mechanisms [58].

Vasculitis is a significant consequence of many primary neuroinflammatory disorders and is also a concerning aspect of secondary inflammatory conditions (such as infections). When present, inflammation causes endothelial damage, thereby altering the vessel wall architecture, resulting in stiff vessels with a loss in vascular reactivity and as a consequence, impairment in cerebral auto-regulatory capacity. Elastography has the ability to measure such changes and has been used in inflammatory conditions such as Bechet’s disease to demonstrate early subclinical vasculitis as suggested by greater vessel stiffness [59]. These early findings may serve to identify subclinical measures of pathologic inflammation sooner, thereby enhancing clinical detection and providing targets for earlier intervention, and prevention of secondary complications.

In summary, ultrasonography, including TCD and elastography, is a non-invasive technique that can provide real-time information about cerebral blood flow, as well as secondary measures of cerebral vasospasm, intracranial pressure, and vascular compliance. All of these measures are significantly impacted during acute and subacute neuroinflammation, are dynamic over the course of the injury and recovery phases, and are critical measures for guiding treatment strategies.

## 4. Electroencephalography (EEG)

EEG is another non-invasive monitoring tool that facilitates evaluation of brain function through measurement of electrical activity and electrical rhythms of the brain. EEG recordings are generated primarily by neural activity arising from groups of cortical pyramidal neurons that are positioned in the cortex perpendicular to the brain surface. Electrodes detect the sum of excitatory and inhibitory postsynaptic potentials from a large number of surrounding neurons, approximately 6 cm^2^ [60]. These potentials represent interconnections between cortical neurons as well as cortical–subcortical connections [60]. Sinusoidal activity is thought to represent oscillatory connections between cortical and subcortical structures, and are generally predominant during resting states. Once the cortex is activated to perform a task, faster lower amplitude activity prevails [60]. During states of brain dysfunction, these normal patterns are not as well visualized and abnormal patterns can arise depending on the underlying pathology. This can provide information about neuronal activity as a result of poor perfusion, oxygen deprivation, and metabolic dysregulation, acknowledging that this is a secondary, and not a direct measure, as brain function is dependent on a plethora of different factors. During inflammatory states, immunometabolic shifts and resultant microglial activation signal cascades to trigger either hyperexcitability or neuronal dysfunction and slowing. Overactive microglia cause neurotoxic effects by producing cytokines that result in ROS and neuron damage, or hyperexcitability and dysfunction by cytokine-induced effects on synaptic transmission, and plasticity though re-modeling ion channel composition and function. EEG patterns as a result are altered and provide information about neuronal function in the face of neuroinflammation [61].

EEG has been a mainstay in NCC since its development, being an important tool largely for monitoring status epilepticus and encephalopathy. It is used for diagnosis and guiding management in status epilepticus, and has been shown to be a good prognostic tool for patient outcomes [62]. In encephalopathy it is used to help quantify the degree of cerebral dysfunction, and some specific characteristics can help aid in diagnosis such as autoimmune encephalitis, and metabolic encephalopathy. Particular wave-forms can be pathognomonic such as triphasic waves in metabolic abnormalities and delta brush in anti-NMDA receptor encephalitis [61]. The underlying pathophysiology behind these findings is unknown, but postulated to be secondary to altered neuronal connectivity with deeper brain structures from neuroinflammation [61]. Animal models of septic encephalopathy have been used to examine the effects of neuroinflammation on brain functioning, and show increased delta frequency (slow waves indicating cerebral dysfunction) and significantly reduced alpha activity (faster waves present during alert states) [63]. There was also a significant correlation between cortical blood flow changes and EEG frequency [63]. Decreased cerebral blood flow and glucose metabolism has been linked to EEG slowing in many prior studies, but whether this is a direct causation on neuronal functioning is unclear [63]. It is possible that the relationship is secondary, and that systemic and local generation of pro-inflammatory molecules causes neuronal slowing. Activated microglia and resultant release of inflammatory mediators, including cytokines, has also been linked to EEG slowing in animal models of sepsis as well as human studies [63].

In summary, EEG affords the opportunity to measure direct neuronal functioning as a result of underlying immunometabolic stability and inflammatory changes. Its integral role neurocritical care monitoring makes it an ideal modality to further examine secondary effects of neuroinflammation and provide early markers of neuronal dysfunction.

## 5. Magnetic Resonance Imaging (MRI)/MR Spectroscopy (MRS)

### 5.1. MRI

Neuroimaging is a commonly used diagnostic and monitoring tool in the context of neurocritical care. Current imaging technology allows for easy acquisition in a relatively rapid time frame and in a non-invasive manner. It can be used to detect a variety of findings including those from stroke, TBI, intracerebral hemorrhage, and intracranial hypertension [64]. Paired with other monitoring tools, neuroimaging can provide useful information for accurate diagnosis, intervention, and monitoring in several scenarios. Computed Tomography (CT) is the most frequently used NCC imaging modality, as well as most other areas of neurology. CT uses X-rays transmitted through the body, and scans can be acquired very quickly, usually taking no more than a few minutes [65].

However, in cases where more detailed information is required, particularly with soft-tissue injuries, MRI is used to provide higher resolution images. MRI works by aligning protons in the tissue through the use of strong magnetic fields. Radiofrequency pulses are added, causing protons to resonate. These pulses are then switched off, allowing protons to return to their resting state. When this happens, signals are emitted, and this produces MR images with highly detailed structural integrity [66]. Although it generally takes a longer time to produce images using MRI, the acquired information provides much greater detail than a CT.

Another advantage of using MRI, is that the acquired information can go beyond visualizing structural components of the brain. Various MRI protocols can also provide information on functional response and neurometabolic changes post-injury [67]. For example, functional MRI (fMRI) can be used to assess changes in blood flow and oxygenation within the brain with high spatial and temporal resolution. Images generated from the hemodynamic response can be induced by a specific sensory task (e.g., finger tapping) but can also be observed in the brain’s “resting state”, without any assigned tasks (resting state fMRI). These tasks are designed to affect a change in neural activity, which leads to dynamic changes in cerebral blood flow (CBF), and the blood oxygen concentration (termed blood oxygen level dependent imaging or BOLD imaging) in the area of interest [68]. fMRI measures these changes and allows for the visualization of activity in the brain directly resulting from the task or from rest. This technique has many clinical applications and if paired with another assessment tool, can give critical information needed for a wide array of assessments including injury localization, pharmacokinetics, and neurosurgical procedures [69]. Arterial Spin Labeling (ASL) is another example, and it is an MRI technique that looks at tissue perfusion by measuring CBF. It uses magnetically-tagged water protons in arterial blood to trace its flow through tissues [70]. This technique is useful for detecting various injuries and diseases including stroke, tumors, infections, hypoxia, and inflammation [71]. The versatility of MRI makes it useful for detecting both acute and secondary injury, as well as underlying causes.

The use of MRI does come with certain limitations, however. Changes over time can only be tracked with sequential scans and cannot be tracked continuously in real time. Tracking the progression of, and recovery from, an injury or disease requires multiple imaging sessions to be conducted, in order for any comparisons to be made. This type of assessment can be difficult to acquire, is expensive, and is not always available in every facility. Additionally, the quality, and therefore utility of MRI scans can be highly influenced by the strength of the magnet, as well as the experience of the user as scan resolution decreases with lower magnet strength. Again, this is facility-dependent which also makes it difficult when a patient is being treated at multiple centers with differing imaging capabilities. Finally, MRI is not suitable for every individual, particularly those who have unstable conditions and/or are critically ill. It can be harmful to patients who continuously require metal equipment (e.g., oxygen tanks) or who have metal implants or leads.

### 5.2. Magnetic Resonance Spectroscopy (MRS)

**Figure 2:**
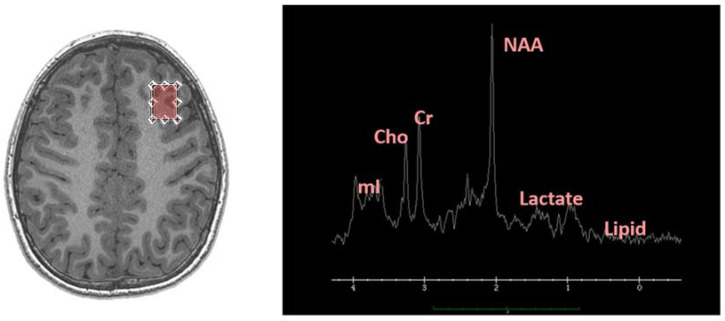
The left panel shows an axial slice of a T1-weighted anatomical scan of a symptomatic mTBI patient generated from a 3.0T scanner. The box indicates the dorsolateral prefrontal cortex (DLPFC) prescription, the selected single-voxel region of interest (ROI) where Magnetic resonance spectroscopy (MRS) data are collected from. The right panel is the MRS output from the same patient. The different peaks are labeled and show relative concentrations of corresponding metabolites.

MRS can be added to an MRI protocol and is typically performed at the end of a scan. MRS can detect the biochemical makeup of a defined area, or region of interest (ROI), of the brain. The most commonly used signals used to study the chemical environment is from hydrogen protons. The region of interest is targeted with the help of MR anatomical scans acquired in the same sitting, and the relative distribution of electrons in the atomic makeup of different molecules allows measurement of different resonance frequencies and magnetic field differentiation [72]. Relative concentrations of important metabolites including choline (Cho; a cell membrane marker), creatine (Cr; an ATP marker), N-acetylaspartate (NAA; a marker of neuronal health), myo-Inositol (mI; a marker of glial health), glutamate and glutamine (Glu; a cellular metabolism marker, and the most abundant excitatory neurotransmitter), lactate (Lac; an anaerobic metabolism marker), and lipids. Further with higher field strength magnets, MRS can detect GABA levels [73,74]. The output is a graph illustrating quantifiable chemical peaks created by the presence of these metabolites [72] (Figure 2). Abnormalities in these peaks can help diagnose the presence of various conditions including brain tumors, metabolic impairment particularly in HIE and TBI, demyelination, and focal infections [72]. For example, elevated levels of cortical and hippocampal Lac and decreased NAA, Glu, Cr, and Cho in the same regions have been shown in TBI after 1 h [75]. These changes can be detected in regions of the brain that structurally appear to be uninjured. These measurements can also be repeated serially to determine how the biochemical environment is changing over time, as well as look at disease and injury prognosis. MRS metabolites can give information about overall cellular health and injury resultant of immune-metabolic dysregulation. Pairing it with another tool to detect inflammatory markers is necessary however for more robust and conclusive results. Looking at the metabolites alone, and in a restricted amount of cortical tissue/space, is not sufficient to determine specific changes in the neuroinflammatory cascades after injury [73], or the clinical impact that these could have on patients.

Single-voxel spectroscopy (SVS) techniques are common with MRS, where the volume of interest (VOI) is generated from a single, rectangular ROI localized on the anatomical scan (Figure 2). This is the most frequently used MRS protocol because it is quick, simple, and relatively easy to acquire, but the limited volume from where information is sourced is not appropriate for all cases. Multi-voxel spectroscopy (MRSI) allows for broader coverage because it can measure metabolites from multiple voxels and multiple slices at the same time [74], thereby providing a more comprehensive evaluation. Metabolite concentrations can be generated from both 2D and 3D images with MRSI. This is helpful when global metabolite patterns are being studied, or when regions of interest are small but irregularly shaped. One disadvantage or MRSI is that it takes longer to acquire scans and the associated spectra, making it less than ideal for clinical application. Fast MRSI techniques have been developed however, and these techniques aim to improve the efficiency of data collection without sacrificing spatial resolution [76].

## 6. Biological Markers—Cytokine Analysis

Cytokines are a diverse set of proteins involved in multiple biological processes and signaling cascades. When released, stimulated by anything from infection to the natural process of aging, they elicit responses largely by binding to receptors on their target cells. They are categorized according to their function and they are currently divided into six families: interferons (involved in antiviral activity), interleukins (involved in regulating immune function), chemokines (involved in cell migration), mesenchymal growth factors (involved in cell renewal), tumor necrosis factors (involved in cell proliferation, differentiation, and apoptosis), and adipokines (involved in metabolism) [77]. In addition to these families, cytokines can also be grossly separated into pro-inflammatory and anti-inflammatory groups [78]. Cytokine analysis is becoming increasingly utilized in an effort to gain a better understanding of biological processes and help guide treatment strategies [1,79,80,81,82].

The use of cytokine analysis is not currently part of standard NCC testing procedures; however, its use in research is becoming more common, especially where neuroinflammation is a large factor in injury pathophysiology and where it can lead to secondary and/or chronic injuries later. This includes TBI, neonatal HIE, stroke and autoimmune encephalitis. Typically, samples of serum, plasma or whole blood are collected and used to detect levels of different cytokine families. These samples are then subjected to either an enzyme-linked immunosorbent assay (ELISA), an antigen-based detection process, or a multiplex array, which allows for detection of multiple cytokines on the same sample [78]. These assays serve to quantify circulating cytokine concentrations, which can aid in the creation of profiles or inflammatory signatures to unearth, or shed light on, patterns about inflammatory and metabolic responses in the body. However, analysis of serum or plasma cytokine levels can be confounded by peripheral factors, as the blood is circulating through the body and various organs, which may also be injured or inflamed and therefore contribute to the signal. Tissue samples can yield more accurate results, but collection is not always possible or appropriate, especially with human patients, and particularly when trying to understand inflammation of the brain where CSF is ideal but often not available.

Using TBI as an example, previous research has shown that cytokine concentrations are temporally and regionally variable in different animal models and in human samples [83,84,85]. Chemokines and cytokines are generally upregulated after injury, and these alterations can be detected from minutes to several weeks and sometimes months after the initial insult. As a result, cytokines are thought to be key players in the processes of secondary injury, and potentially recovery, that occurs after TBI. As such they have been hypothesized as potential targets for diagnosis, prognosis, and potential interventions to lessen the symptom burden during this phase or enhance recovery processes. For example in neonates with HIE, levels of several interleukins, TNF-α, and IFN-γ are also elevated and are associated with injury severity and outcome [81]. Similarly, in patients with stroke, IL-1β, IL-6, and TNFs have been shown to increase after injury [86]. Interestingly, comparison of these types of studies also reveal that no single biomarker can fully explain the extent, evolution, effect of treatment, or prognosis of an injury [6,87,88,89]. Investigating multiple cytokines and cytokine families, over time, therefore strengthens the reliability of using these molecular markers as diagnostic and prognostic tools. As with MRS, cytokine analysis can benefit from being compared to data collected using other metrics. Comparisons between multiple modalities can help reveal patterns across different individuals and identify dynamic, personalized treatment and management strategies [90,91].

## 7. Rapid Whole Exome Sequencing (WES) and RNA Sequencing

Whole exome sequencing is not generally thought of as an acute tool used in the NCC setting and does not provide continuous monitoring of ongoing brain function. However, management strategies are becoming more and more complex, and a better appreciation of susceptibility modifiers and individualized responses are essential to improve patient outcomes. The importance of genetic modifiers of risk and resilience cannot be overstated as they can dramatically alter the physiological response to injury, as well as impact treatment response [92]. With the advent of whole exome testing (WES) a new tool is available that can provide more individualized data to guide bedside decision making. Research has shown that by providing rapid processing of WES, information gained helped decrease infant morbidity from changing clinical care decisions, as well as reduced cost of hospitalization by greater than 50% due to limiting unnecessary investigations [93,94]. More recent studies have shown the same benefit in pediatric ICUs, demonstrating changes to clinical management in terms of pharmacology, goals of care, and invasive treatments, to provide better patient outcomes [95,96].

WES used in tandem with multi-modal bedside monitoring can help with interpretation of data and guide treatment with various aspects of management including pharmacotherapy. As mentioned in the paragraphs above, the goal of multi-modal monitoring is to be able to measure patient changes from a systems biology perspective in order to make adjustments to treatment. Bedside monitoring measures both direct and indirect markers of metabolism, and interpretation of these markers is dependent on individual metabolic profile. For instance, patients with acute inflammatory conditions may have mutations in metabolic pathways of immune cells that could underlie an abnormal inflammatory response [97]. This in turn may alter the metabolic and immune profile monitored at the bedside, which can change interpretation of the data. As an example, data examining patients with mitochondrial disease using NIRS showed lower variability in tissue oxygenation index at baseline compared to control patients [98,99]. While this was in skeletal muscle, there is potential to translate this technique into neurological tissue energetics. This information would be crucial to know in order to accurately interpret NIRS data collected at bedside in patients with mitochondrial diseases and make rapid clinical decisions. Additionally, pharmacological immunotherapy used in neuroinflammatory syndromes depends on a presumption of intact metabolism and function of the various modulating cells. Research has demonstrated cases of critically ill patients with adverse outcomes due to drug toxicity from unknown underlying mutations in drug metabolism pathways, such has the CYP450 enzymes [100,101]. The importance of this is paramount, as >90% of the drugs used in ICU settings have some component of CYP450 metabolism [102]. This is compounded by the fact that in critical illness, and with children in general, the drug pharmacokinetics and dynamics may be different than what is expected outside of this setting due to many factors including the etiology itself, blood perfusion, end organ injury, other medications, and genetic predisposition. In this context WES results on susceptibility or resilience modifiers could prove very valuable in individualized acute care pathways.

New research is shedding light on genetic variability having a modulatory effect on the neuroinflammatory response. Evidence from studies of single nucleotide polymorphisms (SNPs) using RNA sequencing have shown the ability to provide information on time-specific gene expression that changes in response to environmental factors. For example, research has shown that in acute inflammatory settings, between 3000 and 5000 genes, up to 20% of the genome, are activated [103]. Additionally, the genes that are activated change over time during an inflammatory response. Sequential sampling can map such genomic expression over time and provide direct targets for pharmacological therapy in order to amplify or inhibit certain genes and alter the inflammatory trajectory. While most research to date has been done in psychiatric and neurodegenerative disorders, early studies are demonstrating evidence of relevant genetic variance in ICU settings [103]. This hints at the possibility of answering the question as to why some people recover from the effects of injury/insult and neuroinflammation but others are left with debilitating injuries, a protracted course of a persistent inflammatory state, or even death. Specifically, SNPs can be used as a biomarker for disease, and for prognostication of risk, outcomes, and treatment response, especially when looking at genes responsible for mounting an inflammatory response in critically ill children. In acute respiratory distress syndrome (ARDS), a systemic inflammatory respiratory disease, SNPs have been identified that are associated with disease severity [104]. Examining acute trauma patients in the Glue Grant Experience [105] revealed that 40% of new peptides and proteins after injuries are likely self-antigens, likely triggering a harmful autoimmune response [106]. These studies highlight the possibility that this information could shed light on individualized disease pathophysiology, and potentially help guide therapeutic advances.

Personalized data regarding genomic transcription and translation is a novel and expanding area of research that provides a better understanding of individual risk from injury and potentially treatment, and can serve to guide pharmacological management, as well as interpret data from bed-side monitoring tools. Previously thought to only be beneficial in chronic diseases, new research is shedding light on its utility in real-time critical care settings of inflammatory diseases to improve patient outcomes [107].

## 8. Conclusions

**Figure 3:**
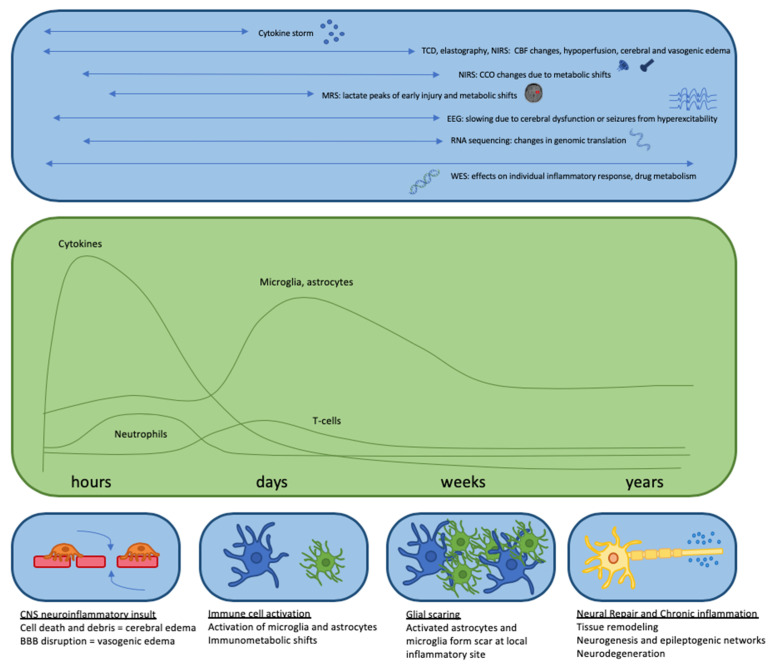
Time course depicting cellular CNS response to neuroinflammation and activation of immune cells, with timing of multi-modal monitoring. Top panel indicates different modes of monitoring during each stage of the CNS inflammatory response, and markers than can be measured. Middle panel indicates activation of different immune cells over time in response to a neuroinflammatory stimuli. Bottom panel indicates cellular and molecular changes over time.

Research evaluating the utility of non-invasive bedside monitoring continues to expand. The ability to use these tools simultaneously provides the opportunity to monitor multiple measures of overall brain health during critical illness and evaluate the effects of biological (normal and pathological) responses, such as inflammation (Figure 3). They provide an extension of the clinical bedside assessment and give insight into an impending or evolving secondary injury to better identify the windows of opportunity for tailored intervention. This is particularly important in a population where a thorough physical exam is not always possible. Additionally, response to treatment can be monitored to better predict positive and negative responses as well as adverse effects, thereby facilitating adjustments in real time. Lastly, these tools may be better able to help prognosticate and guide decision making for patients and their families.

In short, multi-modal monitoring affords the ability to guide choice of treatment in neurological conditions, and in particular conditions that result in neuroinflammation and immunometabolic dysregulation affecting brain function. As discussed at length in this review article, CNS immunometabolism is an emerging area of research that is providing information regarding mechanistic underpinnings of pathologic neuroinflammation. Multimodal monitoring has the capability of guiding immunotherapy in terms of aggressiveness and timing of treatment, by detecting secondary measures of immunometabolism contributing to the degree of inflammation. A shortfall of current management is the broad degree of suppression that traditional immunomodulation causes, inhibiting both beneficial and detrimental mediators. A classic example of this is corticosteroids, which inhibit an extensive range of inflammatory targets. New therapies are being developed that can target specific metabolic intermediates, or immune pathways, to change from a pro-inflammatory to quiescent, or anti-inflammatory phenotype rather than inhibiting cell function altogether [108,109]. By using these more targeted approaches to treat neuroinflammatory disease, hopefully this will lead to ongoing activity of beneficial inflammation, and inhibition of detrimental factors. This ultimately will lead to better patient outcomes.

Multi-modal monitoring is a way of using non-invasive bedside techniques to measure and monitor secondary markers of immunometabolism in an effort to guide neuroinflammatory treatments and potentially more targeted biologics. This technique is at the forefront of clinical monitoring and management of neuroinflammatory disease due to its ability to discern underlying pathophysiology, track individualized biomarkers over time, and guide targeted therapies. With ongoing research, multimodal monitoring could prove to be a fundamental tool at the forefront of neurocritical care.
